# Supplementary material for: Influence of Dietary Polyunsaturated Fatty Acid Intake on Potential Lipid Metabolite Diagnostic Markers in Renal Cell Carcinoma: A Case-Control Study
Source: Nutrients. 2024 Apr 24;16(9):1265. doi: 10.3390/nu16091265 (PMC11085891; doi:10.3390/nu16091265)
Supplement: Supplementary file 1 [file nutrients-16-01265-s001.zip › Supplementary Materials.pdf]

Table S1. Distribution of cancer diagnoses, sites, and stages at diagnosis of renal cell carcinoma.

Diagnosis		Total (*n*=87)	Discovery set (*n*=60)	Validation set (*n*=27)
Renal cell carcinoma	Clear cell type	80 (92.0)	57 (95.0)	23 (85.2)
	Chromophobe type	4 (4.6)	1 (1.70)	3 (11.1)
	Papillary type	2 (2.3)	1 (1.70)	1 (3.70)
	Unclassified	1 (1.1)	1 (1.70)	0 (0.00)
Cancer site				
Kidney, right		48 (55.2)	32 (53.3)	16 (59.3)
Kidney, left		39 (44.8)	28 (46.7)	11 (40.7)
T stage				
1a		62 (71.3)	43 (71.7)	19 (70.4)
1b		6 (6.9)	4 (6.70)	2 (7.40)
2a		5 (5.7)	5 (8.30)	0 (0.00)
2b		2 (2.3)	1 (1.70)	1 (3.70)
3a		12 (13.8)	7 (11.7)	5 (18.5)
Pathology stage				
N0		10 (11.5)	8 (13.3)	2 (7.4)
Nx		77 (88.5)	52 (86.7)	25 (92.6)
Radical/Partial				
Radical		20 (23.0)	14 (23.3)	5 (18.5)
Partial		67 (77.0)	46 (76.7)	22 (81.5)
Fuhrman nuclear grade				
1		1 (1.1)	1 (1.70)	0 (0.00)
2		47 (54.0)	33 (55.0)	14 (51.9)
3		37 (42.5)	25 (41.7)	12 (44.4)
4		2 (2.3)	1 (1.70)	1 (3.70)
Tumor size				
Longest diameter (cm)		3.57 (3.08)	3.41 (2.50)	3.94 (4.12)
Width (cm)		3.57 (3.08)	3.41 (2.50)	3.94 (4.12)
Length (cm)		2.96 (2.70)	2.79 (1.99)	3.35 (3.87)
High (cm)		2.39 (1.86)	2.38 (1.64)	2.42 (2.33)
Margin(Only partial nephrectomy)				
Surgical margin (negative)		67 (100.0)	46 (100.0)	21(100.0)$
Safety margin (cm)		0.15 (0.13)	0.14 (0.08)	0.17 (0.19)
Necrosis				
Tumor necrosis		48 (55.2)	33 (55.0)	15 (55.6)
Necrosis rate %		14.5 (20.5)	11.2 (15.1)	21.9 (28.3)
Existence of cancer invasion				
Renal capsule		41 (47.1)	27 (45.0)	14 (51.9)
Perirenal fat		3 (3.4)	2 (3.30)	1 (3.70)
Renal sinus fat		4 (4.6)	3 (5.00)	1 (3.70)
Lymphatic		0 (0.0)	0 (0.00)	0 (0.00)
Venous		7 (8.0)	4 (6.70)	3 (11.1)
Perineural		0 (0.0)	0 (0.00)	0 (0.00)
Collecting system		20 (23.0)	13 (21.7)	7 (25.9)

Data are presented as mean (standard deviation) for continuous variables and n (%) for categorical variables.

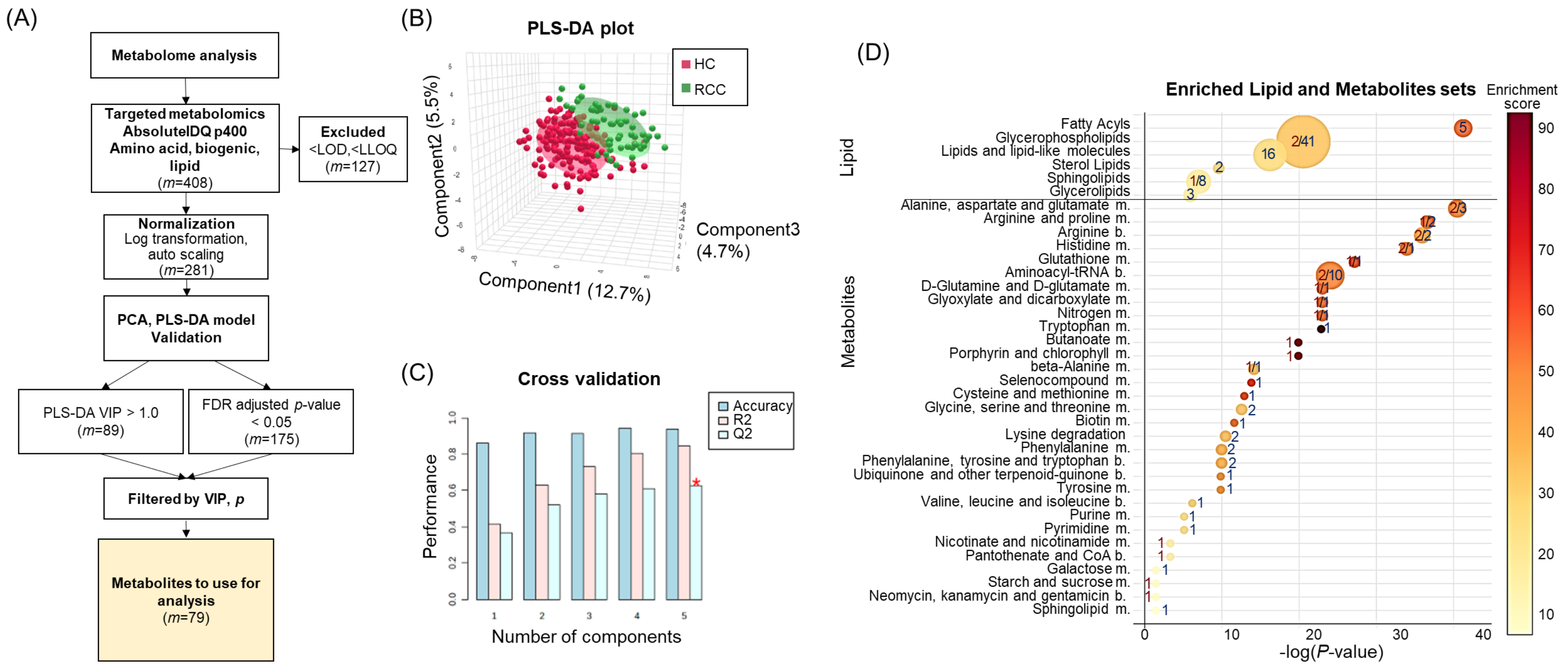

Figure S1. Metabolite data validation and principal enrichment, along with the network of 79 metabolites
(A) Flow chart of metabolomics analysis. (B) PLS-DA plot between healthy control(HC) and Renal cell carcinoma (RCC) groups using all metabolites. (C) Accuracy, R2, and Q2 of Ten-fold cross-validation. (D) Enrichment analysis based on lipid and metabolite metabolism. The horizontal axis represents the negative logarithm of the p-value for enrichment. The size of each circle represents the number of hits for the corresponding lipid or metabolism. An increase in the relative abundance in RCC compared to that in HC is indicated by a red number, whereas a decrease in RCC is indicated by a blue number. The figure was generated using the Cytoscape CyPlot application. Abbreviation: m; metabolites, b; biosynthesis. Normalization by QC sample, log transformation, and autoscaling.

Table S2. Seventy-nine statistically significant metabolites between HC and RCC groups

	Compound name	Short name	CAS	HMDB ID	Pubchem CID	VIP	FDR adjusted p-value	Fold change	log2fold	Class	Updown (RCC /HC)
1	LysoPC(16:0)	LPC(16:0)		HMDB0010382	460602	1.2260	1.50.E-18	0.7133	-0.4874	Lysophosphatidylcholines	Down
2	L-Tryptophan	Trp	73-22-3	HMDB0000929	6305	1.2841	3.04.E-18	0.7468	-0.4212	Amino acids	Down
3	LysoPC(18:0)	LPC(18:0)		HMDB0010384	497299	1.2599	4.69.E-17	0.6856	-0.5446	Lysophosphatidylcholines	Down
4	Decanoylcarnitine	AC(10:0)	1492-27-9	HMDB0000651	10245190	2.9754	1.73.E-16	0.3276	-1.6100	Acylcarnitines	Down
5	LysoPC(18:2(9Z,12Z))	LPC(18:2)	22252-07-9	HMDB0010386	11005824	1.5542	8.46.E-16	0.6366	-0.6515	Lysophosphatidylcholines	Down
6	PC(16:0/20:2(11Z,14Z))	PC(36:2)		HMDB0007979	52922420	1.1484	1.86.E-14	0.7270	-0.4600	Phosphatidylcholines	Down
7	LysoPC(18:1(9Z))	LPC(18:1)	3542-29-8	HMDB0002815	16081932	1.1855	1.34.E-13	0.7116	-0.4908	Lysophosphatidylcholines	Down
8	L-Asparagine	Asn	70-47-3	HMDB0000168	6267	1.1231	3.88.E-13	0.8249	-0.2778	Amino acids	Down
9	L-Alanine	Ala	56-41-7	HMDB0000161	5950	1.1808	3.14.E-12	0.7616	-0.3929	Amino acids	Down
10	L-Glutamic acid	Glu	56-86-0	HMDB0000148	33032	2.0690	1.70.E-11	1.4753	0.5610	Amino acids	Up
11	L-Methionine	Met	63-68-3	HMDB0000696	6137	1.0039	1.58.E-10	0.8135	-0.2977	Amino acids	Down
12	9-Decenoylcarnitine	AC(10:1)		HMDB0013205	53481699	2.7108	1.62.E-10	0.0768	-3.7028	Acylcarnitines	Down
13	LysoPC(20:3(5Z,8Z,11Z))	LPC(20:3)	1199257-41-4	HMDB0010393	53480467	1.3236	1.03.E-09	0.5661	-0.8208	Lysophosphatidylcholines	Down
14	DG(14:0/0:0/18:1n9)	DG(32:1)		HMDB0055962		1.9332	1.33.E-08	0.2793	-1.8402	Diglycerides	Down
15	LysoPC(P-16:0)	LPC-O(16:1)		HMDB0010407	10917802	1.7038	2.52.E-08	0.4337	-1.2053	Lysophosphatidylcholines	Down
16	4-Hydroxyproline	t4-OH-Pro	51-35-4	HMDB0000725	5810	1.3031	3.36.E-08	0.6170	-0.6968	Biogenic amines	Down
17	TG(14:0/14:1(9Z)/16:1(9Z))	TG(44:2)			56937930	1.5862	4.52.E-08	0.2618	-1.9337	Triglycerides	Down
18	L-Octanoylcarnitine	AC(8:0)	25243-95-2	HMDB0000791	11953814	1.8578	7.74.E-08	0.3419	-1.5484	Acylcarnitines	Down
19	LysoPC(17:0)	LPC(17:0)	50930-23-9	HMDB0012108	24779463	1.5984	1.06.E-07	0.4399	-1.1848	Lysophosphatidylcholines	Down
20	TG(14:0/14:0/16:1(9Z))	TG(44:1)		HMDB0042069		1.9045	1.53.E-07	0.4282	-1.2236	Triglycerides	Down
21	DG(18:0/24:1(15Z)/0:0)	DG(42:1)		HMDB0007181	53478056	1.0256	1.58.E-07	0.7031	-0.5082	Diglycerides	Down
22	2-Octenoylcarnitine	AC(8:1)		HMDB0013324	70679121	1.8132	4.02.E-07	0.3187	-1.6496	Acylcarnitines	Down
23	PC(o-18:1(11Z)/18:2(9Z,12Z))	PC-O(36:3)		HMDB0013425	53481721	1.0602	5.97.E-07	0.7606	-0.3948	Phosphatidylcholines	Down
24	Sarcosine	Sarcosine	107-97-1	HMDB0000271	1088	1.6049	8.39.E-07	0.5800	-0.7858	Biogenic amines	Down
25	TG(15:0/18:2(9Z,12Z)/20:3n6)	TG(53:5)			56938956	1.6340	8.71.E-07	0.5994	-0.7384	Triglycerides	Down
26	DG(16:0/0:0/18:3n6)	DG(34:3)				1.7955	1.03.E-06	0.5382	-0.8937	Diglycerides	Down
27	PC(o-16:1(9Z)/18:2(9Z,12Z))	PC-O(34:3)		HMDB0013413	53481709	1.0221	1.08.E-06	0.7554	-0.4047	Phosphatidylcholines	Down
28	SM(d18:1/26:0)	SM(44:1)			44260129	1.4306	1.78.E-06	0.6543	-0.6119	Sphingomyelins	Down
29	PC(o-16:1(9Z)/16:1(9Z))	PC-O(32:2)		HMDB0013411	53481705	1.8205	2.31.E-06	0.2960	-1.7563	Phosphatidylcholines	Down
30	PC(O-16:0/18:2(9Z,12Z))	PC-O(34:2)	88542-95-4	HMDB0011151	6443157	1.0041	2.65.E-06	0.7579	-0.3999	Phosphatidylcholines	Down
31	Butyrylcarnitine	AC(4:0)	25576-40-3	HMDB0002013	213144	1.6043	2.70.E-06	0.4733	-1.0791	Acylcarnitines	Down
32	LysoPC(16:1(9Z))	LPC(16:1)	76790-27-7	HMDB0010383	24779461	1.1224	3.62.E-06	0.7120	-0.4900	Lysophosphatidylcholines	Down
33	sphingomyelin 43:1	SM(43:1)				1.0198	1.15.E-05	0.6813	-0.5536	Sphingomyelins	Down
34	TG(14:0/20:2n6/14:0)	TG(48:2)				1.3485	1.53.E-05	0.6204	-0.6888	Triglycerides	Down
35	TG(16:1(9Z)/14:0/16:1(9Z))	TG(46:2)		HMDB0010419	53480484	1.7581	1.63.E-05	0.5184	-0.9479	Triglycerides	Down
36	DG(16:1n7/0:0/16:1n7)	DG(32:2)				1.3428	2.29.E-05	0.2173	-2.2022	Diglycerides	Down
37	DG(18:1(11Z)/24:1(15Z)/0:0)	DG(42:2)		HMDB0007210	53478085	1.3422	2.38.E-05	0.5918	-0.7569	Diglycerides	Down
38	phosphatidylcholine 43:2	PC(21:0/22:2)				2.0016	4.96.E-05	3.9119	1.9679	Phosphatidylcholines	Up
39	Ceramide (d18:1/22:0)	Cer(40:1)				1.3868	1.62.E-04	0.4585	-1.1249	Ceramide	Down
40	TG(15:0/18:3(6Z,9Z,12Z)/15:0)	TG(48:3)				2.3950	1.98.E-04	0.5679	-0.8164	Triglycerides	Down

41	TG(16:0/22:6(4Z,7Z,10Z,13Z,16Z,19Z)/16:0	TG(54:6)				1.0115	2.06.E-04	0.7800	-0.3585	Triglycerides	Down
42	DG(18:1(11Z)/16:0/0:0)	DG(34:1)			5283470	1.0522	2.26.E-04	0.7375	-0.4394	Diglycerides	Down
43	PC(22:1(13Z)/22:6(4Z,7Z,10Z,13Z,16Z,19Z)	PC(44:7)			52923545	1.1948	2.98.E-04	0.6228	-0.6832	Phosphatidylcholines	Down
44	TG(16:1(9Z)/16:1(9Z)/20:3n6)	TG(52:3)			9544149	1.0132	2.98.E-04	0.7765	-0.3649	Triglycerides	Down
45	SM(d18:1/22:0)	SM(40:1)		HMDB0012103	44260125	1.8136	3.21.E-04	1.2974	0.3756	Sphingomyelins	Up
46	Cer(d18:1/24:1(15Z))	Cer(42:2)		HMDB0004953	5283568	1.1832	5.38.E-04	0.7489	-0.4172	Ceramide	Down
47	TG(15:0/15:0/20:3n6)	TG(50:3)		HMDB0043006		1.1799	5.56.E-04	0.7722	-0.3729	Triglycerides	Down
48	phosphatidylcholine O-37:6	PC-O(37:6)				1.6559	5.59.E-04	0.2905	-1.7834	Phosphatidylcholines	Down
49	TG(14:0/20:1(11Z)/15:0)	TG(49:1)				1.1318	8.21.E-04	0.6985	-0.5176	Triglycerides	Down
50	TG(15:0/18:1(9Z)/22:6(4Z,7Z,10Z,13Z,16Z,19Z))	TG(55:7)			56938947	2.3958	1.56.E-03	0.5326	-0.9089	Triglycerides	Down
51	Methionine sulfoxide	MetO	**3226-65-1**	HMDB0002005	158980	1.2229	1.74.E-03	0.5470	-0.8704	Amino acids	Down
52	TG(16:0/16:1(9Z)/18:1(9Z))	TG(50:2)		HMDB0005377	9544011	1.1446	2.53.E-03	0.7748	-0.3682	Triglycerides	Down
53	TG(15:0/22:2(13Z,16Z)/18:4(6Z,9Z,12Z,15Z))	TG(55:6)				2.4109	2.85.E-03	0.5350	-0.9023	Triglycerides	Down
54	TG(15:0/18:1(9Z)/16:1(9Z))	TG(49:2)				1.5734	2.97.E-03	0.6854	-0.5450	Triglycerides	Down
55	DG(16:0/22:0/0:0)	DG(38:0)		HMDB0007115	9543762	1.2072	2.97.E-03	0.6646	-0.5895	Diglycerides	Down
56	L-Aspartic acid	Asp	56-84-8	HMDB0000191	5960	1.0024	2.97.E-03	1.5470	0.6295	Amino acids	Up
57	TG(14:0/22:2(13Z,16Z)/15:0)	TG(51:2)				1.0802	3.01.E-03	0.7923	-0.3358	Triglycerides	Down
58	DG(16:0/20:4(5Z,8Z,11Z,14Z)/0:0)	DG(36:4)		HMDB0007112	9543736	1.1055	3.16.E-03	0.7941	-0.3327	Diglycerides	Down
59	Linoelaidyl carnitine	**Linoelaidyl carnitine**	85114-47-2	HMDB0006461		1.1124	3.30.E-03	0.4040	-1.3074	Acylcarnitines	Down
60	PC(14:0/18:3(9Z,12Z,15Z))	PC(32:3)		HMDB0007876	24778625	1.3708	3.32.E-03	0.6776	-0.5614	Phosphatidylcholines	Down
61	TG(14:0/22:0/14:1(9Z))	TG(50:1)				1.0962	3.32.E-03	0.7515	-0.4122	Triglycerides	Down
62	TG(14:0/22:4(7Z,10Z,13Z,16Z)/15:0)	TG(51:4)				1.1997	4.19.E-03	0.7958	-0.3295	Triglycerides	Down
63	TG(14:0/16:1(9Z)/20:3n6)	TG(50:4)			56938050	1.1815	6.77.E-03	0.7745	-0.3687	Triglycerides	Down
64	TG(14:0/22:5(4Z,7Z,10Z,13Z,16Z)/15:0)	TG(51:5)				1.3946	7.04.E-03	0.6217	-0.6858	Triglycerides	Down
65	phosphatidylcholine O-32:3	PC-O(32:2)			24779281	1.7791	8.40.E-03	0.6085	-0.7168	Phosphatidylcholines	Down
66	TG(14:0/20:0/14:1(9Z))	TG(48:1)				1.1588	8.51.E-03	0.6585	-0.6027	Triglycerides	Down
67	TG(15:0/16:1(9Z)/22:5(4Z,7Z,10Z,13Z,16Z))	TG(53:6)			56938840	2.1942	9.71.E-03	0.6554	-0.6096	Triglycerides	Down
68	TG(15:0/16:0/20:3n6)	TG(51:3)			53481033	1.1012	1.01.E-02	0.8592	-0.2190	Triglycerides	Down
69	PC(20:0/20:2(11Z,14Z))	PC(40:2)		HMDB0008276	24779046	1.4044	1.03.E-02	0.5200	-0.9434	Phosphatidylcholines	Down
70	PC(20:4(5Z,8Z,11Z,14Z)/24:1(15Z))	PC(44:5)				1.5470	1.06.E-02	0.4422	-1.1771	Phosphatidylcholines	Down
71	O-tridecanoylcarnitine	*O*-tridecanoylcarnitine				1.0703	1.88.E-02	0.3375	-1.5672	Acylcarnitines	Down
72	PC(o-22:0/20:4(8Z,11Z,14Z,17Z))	PC-O(42:4)				1.4274	1.90.E-02	0.6462	-0.6301	Phosphatidylcholines	Down
73	TG(16:0/16:1(9Z)/20:5(5Z,8Z,11Z,14Z,17Z))	TG(52:5)			9544180	1.0188	2.72.E-02	0.7385	-0.4373	Triglycerides	Down
74	PC(15:0/20:0)	PC(35:0)		HMDB0007944	52922322	1.0112	2.99.E-02	1.3061	0.3853	Phosphatidylcholines	Up
75	SM(d18:1/26:1(17Z))	SM(44:1)		HMDB0013461	44260128	1.6295	3.02.E-02	0.7235	-0.4669	Sphingomyelins	Down
76	PC(18:0/15:0)	PC(33:0)		HMDB0008033	52922645	1.0399	3.21.E-02	0.8481	-0.2378	Phosphatidylcholines	Down
77	TG(15:0/16:0/22:4(7Z,10Z,13Z,16Z))	TG(53:4)			56938814	1.1953	3.22.E-02	0.9190	-0.1218	Triglycerides	Down
78	sphingomyelin 33:2	SM(33:2)			52931135	1.1819	3.22.E-02	0.3330	-1.5864	Sphingomyelins	Down
79	PC(22:1(13Z)/24:1(15Z))	PC(46:2)				1.0069	3.45.E-02	0.8088	-0.3061	Phosphatidylcholines	Down

Table S3. Multivariate logistic regression analysis of the relationship between HC and RCC metabolites and dietary fats

Category	Logistic Regression	Case	Control	Log of OR (95%CI)	*p*	Adjusted *p*
Decanoylcarnitine	Continuous scale	60	167	-0.6299 (-0.8121 ~ -0.4477)	1.22e-11	8.57e-11
	Half2(>0.59)	4	83	-1.2329 (-1.7179 ~ -0.7480)	6.26e-07	4.38e-06
	Half1(≤0.59)	56	84	ref		
LysoPC(18:2(9Z,12Z))	Continuous scale	60	167	-0.6905 (-0.9087 ~ -0.4724)	5.51e-10	3.85e-09
	Half2(>0.33)	3	82	-1.2746 (-1.8146 ~ -0.7346)	3.72e-06	2.61e-05
	Half1(≤0.33)	57	85	ref		
LysoPC(16:0)	Continuous scale	60	167	-0.7459 (-0.9763 ~ -0.5156)	2.20e-10	1.54e-09
	Half2(>0.41)	2	82	-1.3905 (-2.0257 ~ -0.7553)	1.78e-05	0.0001
	Half1(≤0.41)	58	85	ref		
L-Glutamic acid	Continuous scale	60	167	0.7111 (0.4783 ~ 0.9439)	2.13e-09	1.49e-08
	Half2(>-0.20)	53	83	1.0011 (0.5966 ~ 1.4057)	1.23e-06	8.62e-06
	Half1(≤-0.20)	7	84	ref		
L-Tryptophan	Continuous scale	60	167	-0.8201 (-1.0728 ~ -0.5675)	1.99e-10	1.39e-09
	Half2(>0.33)	4	82	-1.1069 (-1.5814 ~ -0.6325)	4.82e-06	3.37e-05
	Half1(≤0.33)	56	85	ref		
LysoPC(18:0)	Continuous scale	60	167	-0.6945 (-0.9112 ~ -0.4778)	3.36e-10	2.35e-09
	Half2(>0.43)	4	82	-1.0470 (-1.5171 ~ -0.5768)	1.28e-05	8.93e-05
	Half1(≤0.43)	56	85	ref		
LysoPC(18:1(9Z))	Continuous scale	60	167	-0.6491 (-0.8626 ~ -0.4356)	2.53e-09	1.77e-08
	Half2(>0.30)	6	83	-0.9085 (-1.3135 ~ -0.5035)	1.10e-05	7.70e-05
	Half1(≤0.30)	54	84	ref		
PC(15:1/22:2)	Continuous scale	60	167	0.2141 (0.0387 ~ 0.3895)	0.0167	0.0586
	Tertile3(>0.55)	14	54	0.4853 (-0.0159 ~ 0.9866)	0.0577	0.1155
	Tertile2(-0.32~0.55)	41	57	1.0304 (0.5538 ~ 1.5069)	2.26e-05	0.0002
	Tertile1(<-0.32)	5	56	ref		
PC(21:0/22:2)	Continuous scale	60	167	0.2432 (0.1191 ~ 0.3674)	0.0001	0.0009
	Half2(>-0.41)	19	17	0.6169 (0.2736~0.9602)	0.0004	0.0030
	Half1(≤-0.41)	41	150	ref		
Energy(%EER)	Continuous scale	27	223	-0.0033 (-0.0076 ~ 0.0010)	0.1311	0.3059
	Tertile3(>127.72)	10	75	-0.2029 (-0.5995 ~ 0.1936)	0.3159	0.5054
	Tertile2(94.67~127.72)	3	74	-0.6372 (-1.2050 ~ -0.0695)	0.0278	0.2226
	Tertile1(<94.67)	14	74	ref		
Carbohydrate(%RNI)	Continuous scale	27	223	-0.0019 (-0.0038 ~ -0.0001)	0.0437	0.153
	Tertile3(>309.07)	6	75	-0.4248 (-0.8824 ~ 0.0327)	0.0688	0.1834
	Tertile2(230.72~309.07)	7	74	-0.2703 (-0.7047 ~ 0.1642)	0.2227	0.3564
	Tertile1(<230.72)	14	74	ref		
PUFA(g/1000kcal)	Continuous scale	27	223	0.0647 (0.0092 ~ 0.1202)	0.0223	0.156
	Tertile3(>7.42)	11	75	0.3062 (-0.1869 ~ 0.7994)	0.2236	0.4472

	Tertile2(5.37~7.42)	11	74	0.2112 (-0.2947 ~ 0.7171)	0.4132	0.6611
	Tertile1(<5.37)	5	74	ref		
n-6 PUFA(g/1000kcal)	Continuous scale	27	223	0.7609 (0.2040 ~ 1.3177)	0.0074	0.0519
	Tertile3(>0.48)	15	75	0.4970 (0.0156 ~ 0.9784)	0.043	0.3442
	Tertile2(0.32~0.48)	7	74	0.1544 (-0.3811 ~ 0.6899)	0.572	0.9152
	Tertile1(<0.32)	5	74	ref		
n-3 PUFA(g/1000kcal)	Continuous scale	27	223	0.1554 (0.0615 ~ 0.2492)	0.0012	0.0082
	Tertile3(>2.41)	14	75	0.4327 (-0.0647 ~ 0.9300)	0.0882	0.2351
	Tertile2(1.53~2.41)	8	74	0.1912 (-0.3465 ~ 0.7290)	0.4858	0.7773
	Tertile1(<1.53)	5	74	ref		
Fish & shellfish (kcal/1000kcal)	Continuous scale	27	223	0.0001 (-0.0081 ~ 0.0083)	0.9849	0.9849
	Tertile3(>42.31)	8	75	0.2412 (-0.3079 ~ 0.7902)	0.3893	0.6228
	Tertile2(25.24~42.31)	15	74	0.5592 (0.0509 ~ 1.0675)	0.0311	0.2486
	Tertile1(<25.24)	4	74	ref		

Adjusted for age, sex, BMI, smoking, and drinking. RNII; Recommended nutrient intake, PUFA; Polyunsaturated fatty acid

Table S4. Metrics based on 4 machine learning techniques.

	Random Forest	XGBoost	GBM	LGBM
AUC score	0.9589±0.0105	0.9386±0.0199	0.8386±0.0405	0.9371±0.0243
Accuracy	0.9029±0.0199	0.9000±0.0285	0.8412±0.0270	0.8559±0.0110
Sensitivity	0.9556±0.0416	0.8667±0.0567	0.8222±0.0737	0.9556±0.0416
Specificity	0.8720±0.0574	0.9320±0.0204	0.8560±0.0408	0.8520±0.0371
Recall	0.7556±0.0667	0.8111±0.0831	0.7556±0.0831	0.5889±0.0567
Precision	0.8602±0.0257	0.8118±0.0496	0.6789±0.0388	0.8218±0.0542
F1 score	0.8034±0.0455	0.8097±0.0591	0.7141±0.0569	0.6827±0.0322

XGB, extreme gradient boosting; GBM, gradient-boosted machine; LGBM, light gradient-boosted machine.

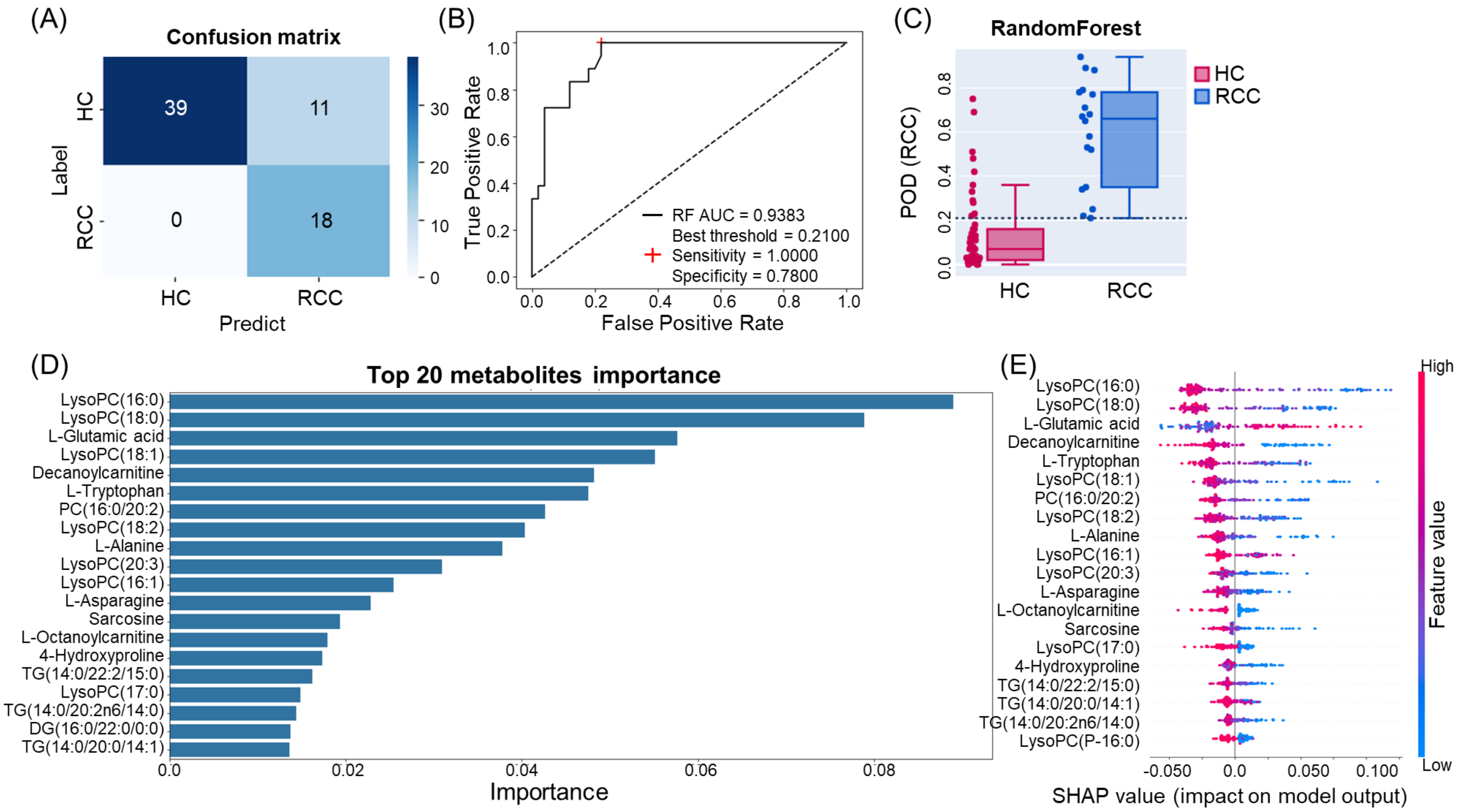

(A)
Confusion matrix
HC
RCC
Label
39
11
0
18
30
20
10
0
HC
RCC
Predict
(B)
True Positive Rate
False Positive Rate
RF AUC = 0.9383
Best threshold = 0.2100
Sensitivity = 1.0000
Specificity = 0.7800
(C)
RandomForest
POD (RCC)
HC
RCC
(D)
Top 20 metabolites importance
LysoPC(16:0)
LysoPC(18:0)
L-Glutamic acid
LysoPC(18:1)
Decanoylcarnitine
L-Tryptophan
PC(16:0/20:2)
LysoPC(18:2)
L-Alanine
LysoPC(20:3)
LysoPC(16:1)
L-Asparagine
Sarcosine
L-Octanoylcarnitine
4-Hydroxyproline
TG(14:0/22:2/15:0)
LysoPC(17:0)
TG(14:0/20:2n6/14:0)
DG(16:0/22:0/0:0)
TG(14:0/20:0/14:1)
Importance
(E)
LysoPC(16:0)
LysoPC(18:0)
L-Glutamic acid
Decanoylcarnitine
L-Tryptophan
LysoPC(18:1)
PC(16:0/20:2)
LysoPC(18:2)
L-Alanine
LysoPC(16:1)
LysoPC(20:3)
L-Asparagine
L-Octanoylcarnitine
Sarcosine
LysoPC(17:0)
4-Hydroxyproline
TG(14:0/22:2/15:0)
TG(14:0/20:0/14:1)
TG(14:0/20:2n6/14:0)
LysoPC(P-16:0)
High
Feature value
Low
SHAP value (impact on model output)

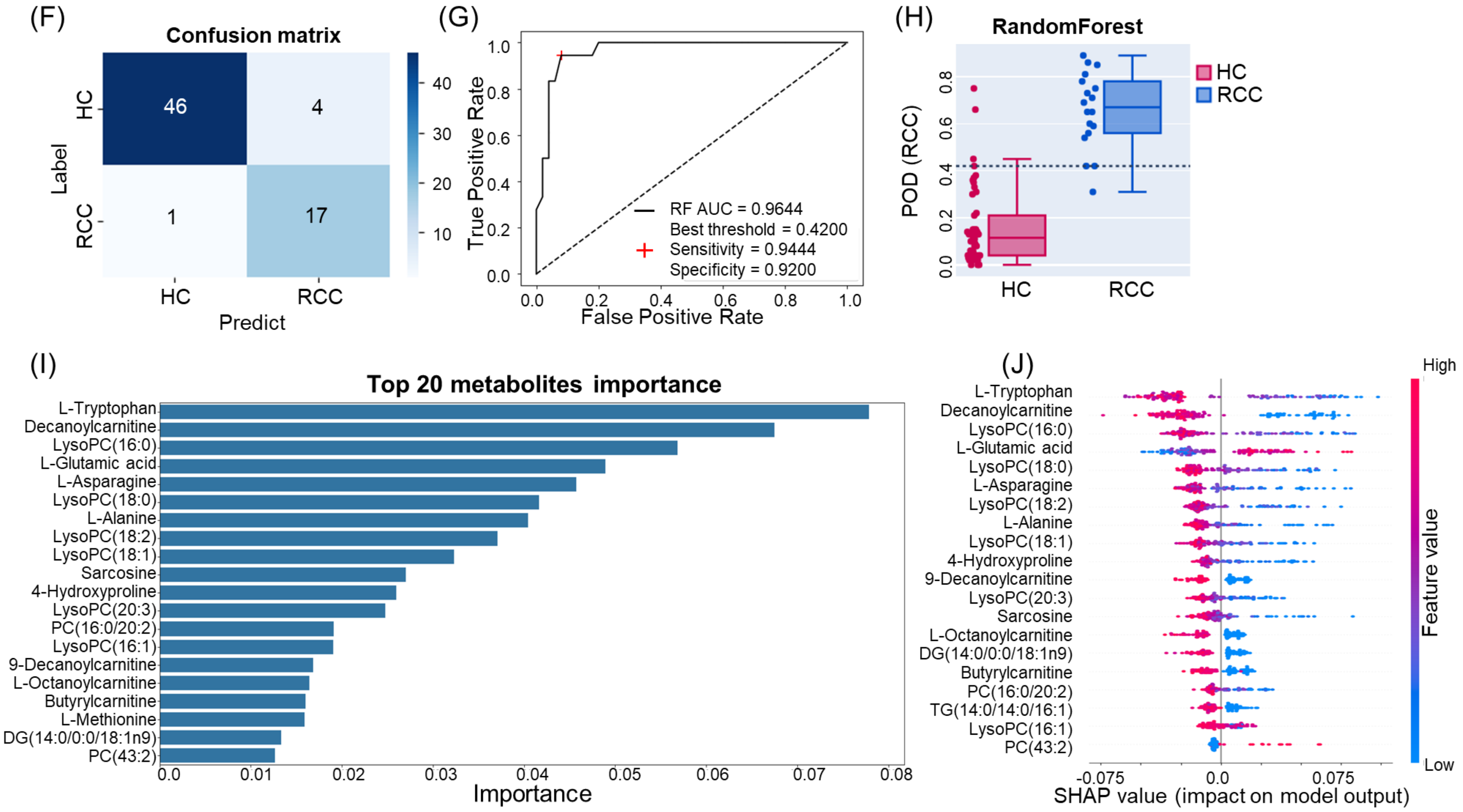

(F)
Confusion matrix
Label
HC
RCC
46
4
1
17
40
30
20
10
HC
RCC
Predict
(G)
1.0
0.8
0.6
0.4
0.2
0.0
True Positive Rate
0.0
0.2
0.4
0.6
0.8
1.0
False Positive Rate
RF AUC = 0.9644
Best threshold = 0.4200
Sensitivity = 0.9444
Specificity = 0.9200
(H)
RandomForest
POD (RCC)
0.0
0.2
0.4
0.6
0.8
HC
RCC
HC
RCC
(I)
Top 20 metabolites importance
L-Tryptophan
Decanoylcarnitine
LysoPC(16:0)
L-Glutamic acid
L-Asparagine
LysoPC(18:0)
L-Alanine
LysoPC(18:2)
LysoPC(18:1)
Sarcosine
4-Hydroxyproline
LysoPC(20:3)
PC(16:0/20:2)
LysoPC(16:1)
9-Decanoylcarnitine
L-Octanoylcarnitine
Butyrylcarnitine
L-Methionine
DG(14:0/0:0/18:1n9)
PC(43:2)
0.0
0.01
0.02
0.03
0.04
0.05
0.06
0.07
0.08
Importance
(J)
L-Tryptophan
Decanoylcarnitine
LysoPC(16:0)
L-Glutamic acid
LysoPC(18:0)
L-Asparagine
LysoPC(18:2)
L-Alanine
LysoPC(18:1)
4-Hydroxyproline
9-Decanoylcarnitine
LysoPC(20:3)
Sarcosine
L-Octanoylcarnitine
DG(14:0/0:0/18:1n9)
Butyrylcarnitine
PC(16:0/20:2)
TG(14:0/14:0/16:1)
LysoPC(16:1)
PC(43:2)
-0.075
0.0
0.075
SHAP value (impact on model output)
High
Feature value
Low

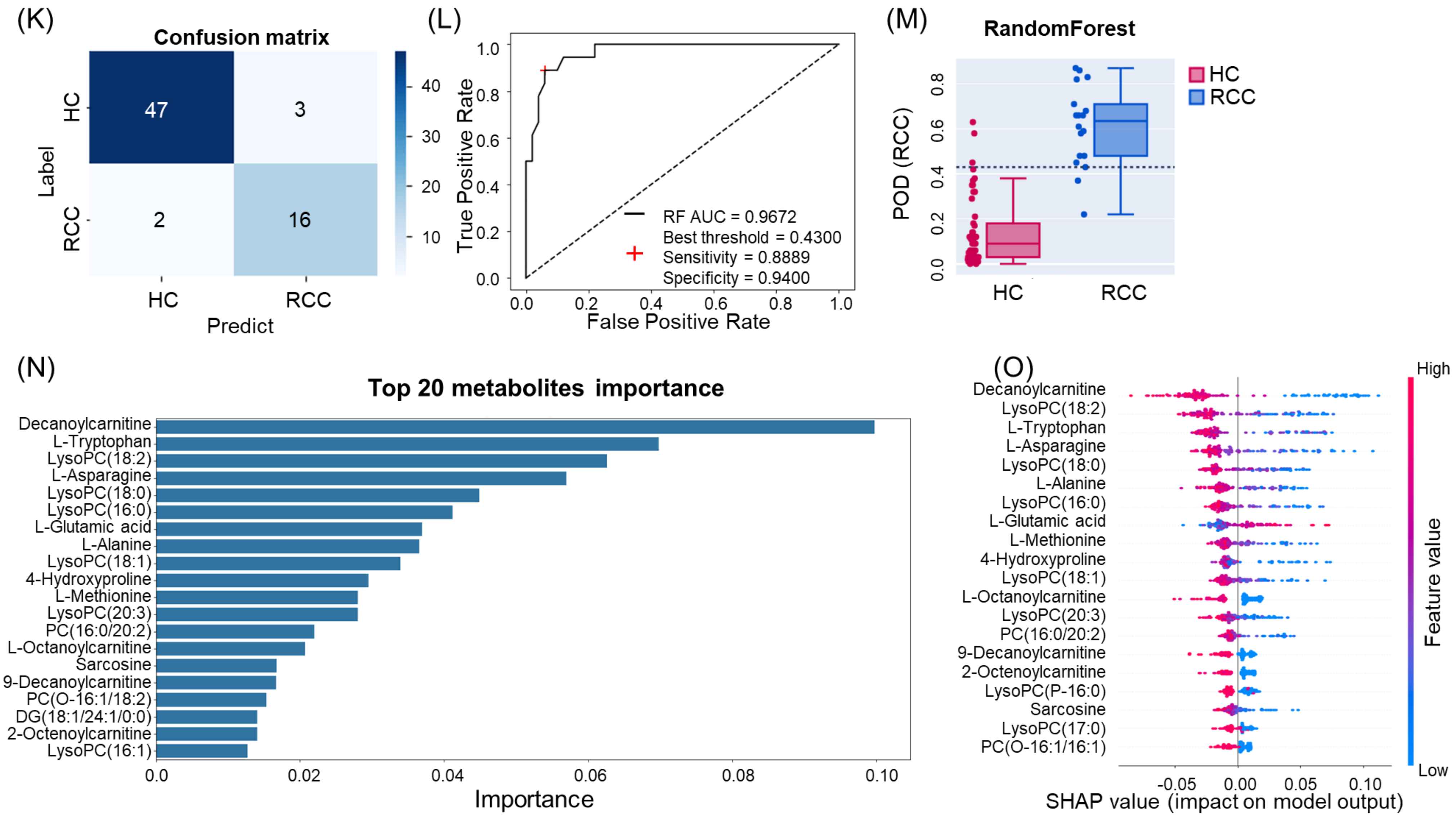

(K)
Confusion matrix
Label
HC
RCC
47
3
2
16
40
30
20
10
HC
RCC
Predict
(L)
True Positive Rate
False Positive Rate
RF AUC = 0.9672
Best threshold = 0.4300
Sensitivity = 0.8889
Specificity = 0.9400
(M)
RandomForest
POD (RCC)
HC
RCC
(N)
Top 20 metabolites importance
Decanoylcarnitine
L-Tryptophan
LysoPC(18:2)
L-Asparagine
LysoPC(18:0)
LysoPC(16:0)
L-Glutamic acid
L-Alanine
LysoPC(18:1)
4-Hydroxyproline
L-Methionine
LysoPC(20:3)
PC(16:0/20:2)
L-Octanoylcarnitine
Sarcosine
9-Decanoylcarnitine
PC(O-16:1/18:2)
DG(18:1/24:1/0:0)
2-Octenoylcarnitine
LysoPC(16:1)
Importance
(O)
Decanoylcarnitine
LysoPC(18:2)
L-Tryptophan
L-Asparagine
LysoPC(18:0)
L-Alanine
LysoPC(16:0)
L-Glutamic acid
L-Methionine
4-Hydroxyproline
LysoPC(18:1)
L-Octanoylcarnitine
LysoPC(20:3)
PC(16:0/20:2)
9-Decanoylcarnitine
2-Octenoylcarnitine
LysoPC(P-16:0)
Sarcosine
LysoPC(17:0)
PC(O-16:1/16:1)
SHAP value (impact on model output)
High
Feature value
Low

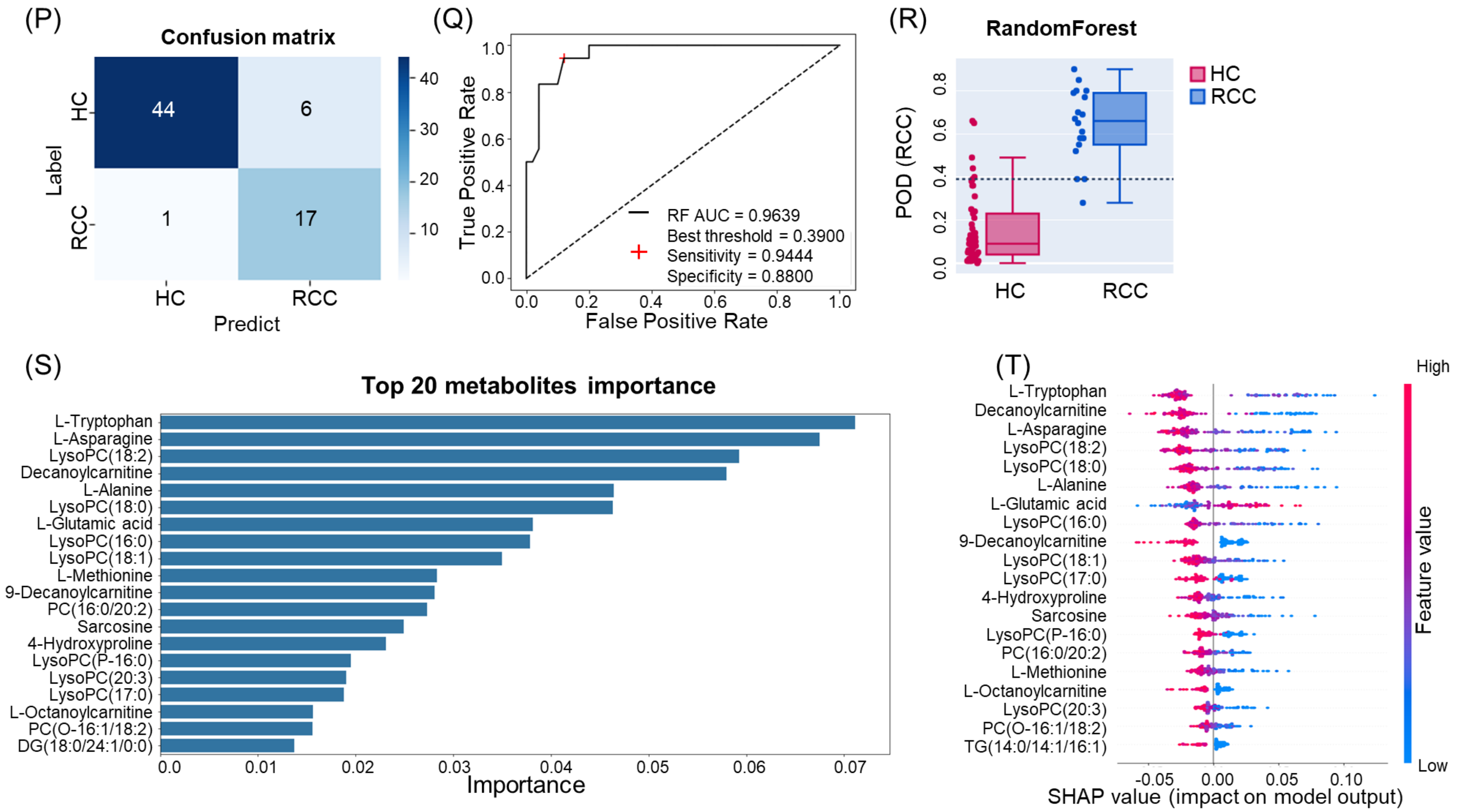
(P)
Confusion matrix
HC
RCC
Label
44
6
1
17
HC
RCC
Predict
40
30
20
10
(Q)
1.0
0.8
0.6
0.4
0.2
0.0
True Positive Rate
RF AUC = 0.9639
Best threshold = 0.3900
Sensitivity = 0.9444
Specificity = 0.8800
0.0
0.2
0.4
0.6
0.8
1.0
False Positive Rate
(R)
RandomForest
POD (RCC)
0.0
0.2
0.4
0.6
0.8
HC
RCC
HC
RCC
(S)
Top 20 metabolites importance
L-Tryptophan
L-Asparagine
LysoPC(18:2)
Decanoylcarnitine
L-Alanine
LysoPC(18:0)
L-Glutamic acid
LysoPC(16:0)
LysoPC(18:1)
L-Methionine
9-Decanoylcarnitine
PC(16:0/20:2)
Sarcosine
4-Hydroxyproline
LysoPC(P-16:0)
LysoPC(20:3)
LysoPC(17:0)
L-Octanoylcarnitine
PC(O-16:1/18:2)
DG(18:0/24:1/0:0)
0.0
0.01
0.02
0.03
0.04
0.05
0.06
0.07
Importance
(T)
L-Tryptophan
Decanoylcarnitine
L-Asparagine
LysoPC(18:2)
LysoPC(18:0)
L-Alanine
L-Glutamic acid
LysoPC(16:0)
9-Decanoylcarnitine
LysoPC(18:1)
LysoPC(17:0)
4-Hydroxyproline
Sarcosine
LysoPC(P-16:0)
PC(16:0/20:2)
L-Methionine
L-Octanoylcarnitine
LysoPC(20:3)
PC(O-16:1/18:2)
TG(14:0/14:1/16:1)
-0.05
0.00
0.05
0.10
SHAP value (impact on model output)
High
Feature value
Low

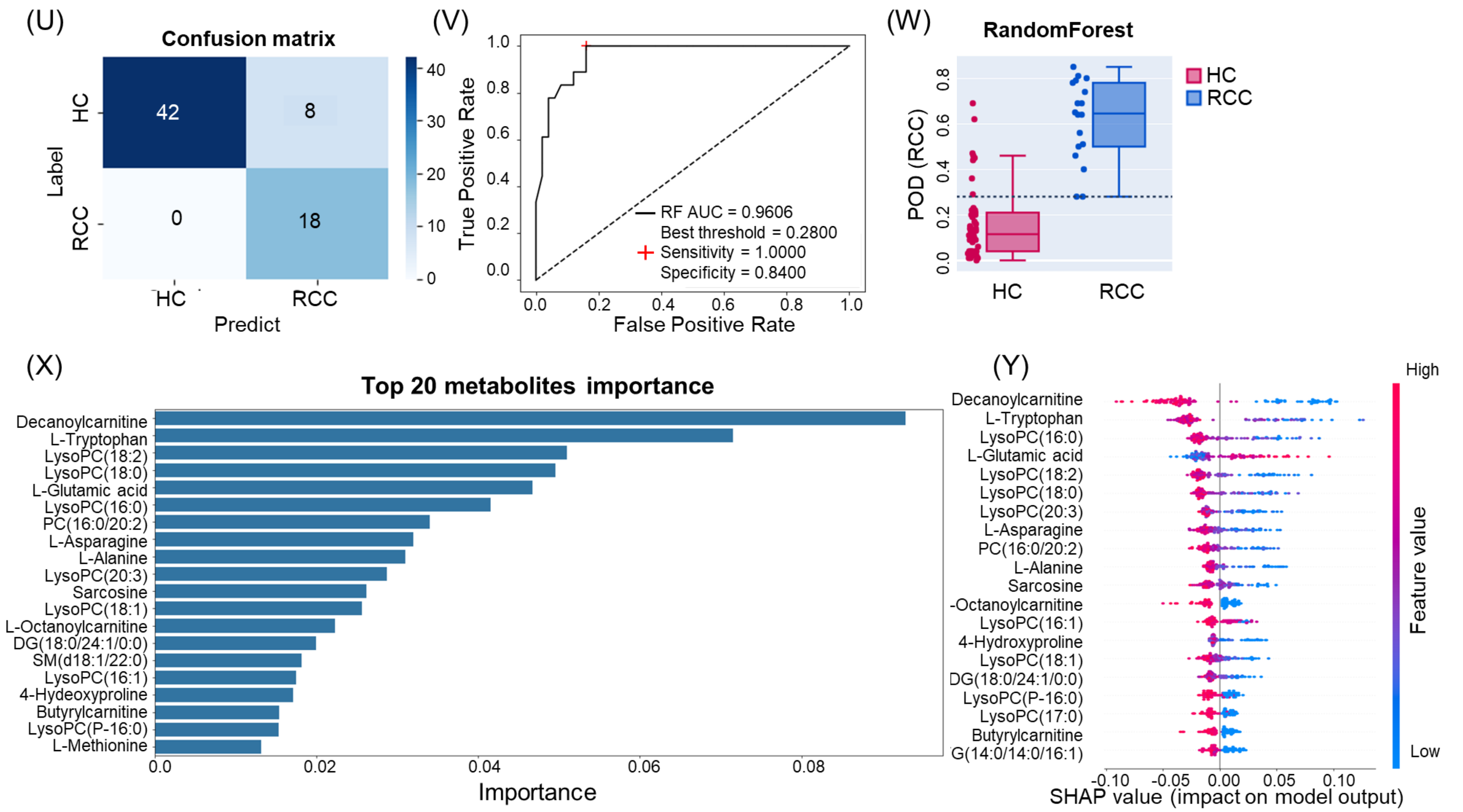

Figure S2. Results of random forest model via machine learning
(A, F, K, P, U) confusion matrix, (B, G, L, Q, V) ROC curve, (C, H, M, R, W) POD plot for each of the five folds , (D, I, N, S, X) Top 20 metabolites importance, (E, J, O, T, Y) SHAP plot.

Table S5. ROC curve analysis of identified potential metabolite markers in RCC

Metabolites	Discovery set		Validation set	
	AUC	*p*	AUC	*p*
L-Tryptophan	0.873	2.16.E-20	0.785	3.92.E-06
LysoPC(16:0)	0.872	5.32.E-21	0.811	9.35.E-08
LysoPC(18:0)	0.858	5.00.E-19	0.784	1.67.E-06
LysoPC(18:2(9Z,12Z))	0.841	1.51.E-17	0.846	1.09.E-08
Decanoylcarnitine	0.823	2.47.E-18	0.869	5.20.E-12
PC(16:0/20:2(11Z,14Z))	0.823	3.98.E-16		
LysoPC(18:1(9Z))	0.820	3.34.E-15	0.791	3.67.E-06
L-Asparagine	0.809	1.10.E-14		
L-Glutamic acid	0.803	6.05.E-13	0.893	4.02.E-11
L-Alanine	0.799	1.01.E-13		
L-Methionine	0.781	8.45.E-12		
4-Hydroxyproline	0.776	2.99.E-09		
LysoPC(20:3(5Z,8Z,11Z))	0.772	6.94.E-11		
LysoPC(P-16:0)	0.762	2.16.E-09		
Sarcosine	0.756	1.16.E-07		
LysoPC(17:0)	0.744	1.17.E-08		
DG(18:0/24:1(15Z)/0:0)	0.743	1.85.E-08		
9-Decenoylcarnitine	0.737	9.25.E-12		
PC(o-16:1(9Z)/18:2(9Z,12Z))	0.733	1.61.E-07		
LysoPC(16:1(9Z))	0.733	6.44.E-07		
PC(o-18:1(11Z)/18:2(9Z,12Z))	0.732	7.65.E-08		
DG(14:0/0:0/18:1n9)	0.725	1.04.E-09		
PC(O-16:0/18:2(9Z,12Z))	0.723	4.43.E-07		
TG(15:0/18:2(9Z,12Z)/20:3n6)	0.720	1.24.E-07		
TG(14:0/14:0/16:1(9Z))	0.709	1.74.E-08		
sphingomyelin 43:1	0.708	2.26.E-06		
DG(18:1(11Z)/24:1(15Z)/0:0)	0.707	5.09.E-06		
L-Octanoylcarnitine	0.707	8.26.E-09		
SM(d18:1/26:0)	0.705	2.85.E-07		
TG(14:0/14:1(9Z)/16:1(9Z))	0.702	4.34.E-09		
TG(14:0/20:2n6/14:0)	0.698	3.05.E-06		
PC(o-16:1(9Z)/16:1(9Z))	0.698	3.78.E-07		
2-Octenoylcarnitine	0.696	5.00.E-08		
TG(16:1(9Z)/14:0/16:1(9Z))	0.688	3.36.E-06		
DG(16:0/0:0/18:3n6)	0.687	1.51.E-07		
DG(18:1(11Z)/16:0/0:0)	0.687	6.76.E-05		
Butyrylcarnitine	0.686	4.61.E-07		
TG(16:0/22:6(4Z,7Z,10Z,13Z,16Z,19Z)/16:0)	0.682	6.05.E-05		
TG(14:0/20:0/14:1(9Z))	0.680	4.27.E-03		
phosphatidylcholine O-37:6	0.677	1.83.E-04		
TG(14:0/16:1(9Z)/20:3n6)	0.675	3.25.E-03		
PC(22:1(13Z)/22:6(4Z,7Z,10Z,13Z,16Z,19Z))	0.671	9.09.E-05		
TG(16:1(9Z)/16:1(9Z)/20:3n6)	0.669	9.13.E-05		
TG(15:0/15:0/20:3n6)	0.666	1.80.E-04		
TG(14:0/22:2(13Z,16Z)/15:0)	0.658	1.25.E-03		
TG(16:0/16:1(9Z)/20:5(5Z,8Z,11Z,14Z,17Z))	0.648	1.55.E-02		
TG(14:0/20:1(11Z)/15:0)	0.648	2.77.E-04		
TG(16:0/16:1(9Z)/18:1(9Z))	0.647	9.91.E-04		
TG(14:0/22:4(7Z,10Z,13Z,16Z)/15:0)	0.646	1.91.E-03		
Ceramide (d18:1/22:0)	0.645	4.21.E-05		
DG(16:1n7/0:0/16:1n7)	0.644	4.80.E-06		
TG(15:0/18:3(6Z,9Z,12Z)/15:0)	0.642	5.64.E-05		
TG(14:0/22:0/14:1(9Z))	0.639	1.43.E-03		
TG(15:0/18:1(9Z)/22:6(4Z,7Z,10Z,13Z,16Z,19Z))	0.633	5.57.E-04		
DG(16:0/20:4(5Z,8Z,11Z,14Z)/0:0)	0.633	1.33.E-03		
TG(15:0/18:1(9Z)/16:1(9Z))	0.630	1.23.E-03		
SM(d18:1/22:0)	0.629	1.01.E-04		

TG(15:0/16:0/20:3n6)	0.628	5.13.E-03		
Cer(d18:1/24:1(15Z))	0.626	1.72.E-04		
Methionine sulfoxide	0.626	6.49.E-04		
PC(14:0/18:3(9Z,12Z,15Z))	0.625	1.44.E-03		
DG(16:0/22:0/0:0)	0.625	1.22.E-03		
TG(15:0/22:2(13Z,16Z)/18:4(6Z,9Z,12Z,15Z))	0.624	1.15.E-03		
L-Aspartic acid	0.619	1.22.E-03		
PC(20:0/20:2(11Z,14Z))	0.618	5.29.E-03		
phosphatidylcholine 43:2	0.618	1.15.E-05		
PC(18:0/15:0)	0.617	1.91.E-02		
PC(15:0/20:0)	0.617	1.73.E-02		
PC(22:1(13Z)/24:1(15Z))	0.614	2.10.E-02		
PC(20:4(5Z,8Z,11Z,14Z)/24:1(15Z))	0.603	5.49.E-03		
TG(14:0/22:5(4Z,7Z,10Z,13Z,16Z)/15:0)	0.603	3.41.E-03		
phosphatidylcholine O-32:3	0.599	4.18.E-03		
TG(15:0/16:1(9Z)/22:5(4Z,7Z,10Z,13Z,16Z))	0.597	4.91.E-03		
SM(d18:1/26:1(17Z))	0.595	1.76.E-02		
Linoelaidyl carnitine	0.595	1.41.E-03		
PC(o-22:0/20:4(8Z,11Z,14Z,17Z))	0.590	1.03.E-02		
sphingomyelin 33:2	0.585	1.93.E-02		
O-tridecanoylcarnitine	0.577	1.01.E-02		
TG(15:0/16:0/22:4(7Z,10Z,13Z,16Z))	0.559	1.93.E-02		

AUC, area under curve

Table S6. Multivariate logistic regression analysis of the relationship between metabolites of HC and T stage 1 RCC

Category	Logistic Regression	Case	Control	Log of OR (95%CI)	*p*	Gg
LysoPC (16:0)	Continuous scale	68	223	-0.04 (-0.05 ~ -0.03)	1.90×10^{-12}	1.33×10^{-11}
	Tertile3(>104.00)	2	75	-1.46 (-2.10 ~ -0.82)	8.14×10^{-06}	3.25×10^{-05}
	Tertile2(88.10-104.00)	5	73	-1.04 (-1.47 ~ -0.62)	1.67×10^{-06}	1.33×10^{-05}
	Tertile1(<88.10)	61	75	ref		
L-Tryptophan	Continuous scale	68	223	-0.06 (-0.08 ~ -0.04)	3.67×10^{-12}	2.57×10^{-11}
	Tertile3(>73.00)	3	75	-1.41 (-1.97 ~ -0.85)	7.00×10^{-07}	5.60×10^{-06}
	Tertile2(63.10-73.00)	5	73	-1.09 (-1.53 ~ -0.66)	8.73×10^{-07}	3.49×10^{-06}
	Tertile1(<63.10)	60	75	ref		
L-Glutamic acid	Continuous scale	68	223	0.02 (0.02 ~ 0.03)	9.48×10^{-12}	6.64×10^{-11}
	Tertile3(>61.80)	62	75	1.72 (1.04 ~ 2.40)	6.66×10^{-07}	5.33×10^{-06}
	Tertile2(44.60-61.80)	4	74	0.39 (-0.38 ~ 1.16)	0.3164	0.5062
	Tertile1(<44.60)	2	74	ref		
LysoPC(18:2(9Z,12Z))	Continuous scale	68	223	-0.55 (-0.72 ~ -0.39)	2.15×10^{-11}	1.51×10^{-10}
	Tertile3(>5.46)	1	75	-1.81 (-2.70 ~ -0.92)	6.80×10^{-05}	0.0003
	Tertile2(4.19-5.46)	11	73	-0.72 (-1.05 ~ -0.38)	2.66×10^{-05}	0.0002
	Tertile1(<4.19)	56	75	ref		
LysoPC(18:0)	Continuous scale	68	223	-0.15 (-0.19 ~ -0.10)	5.26×10^{-11}	3.68×10^{-10}
	Tertile3(>20.80)	2	74	-1.38 (-2.01 ~ -0.74)	2.14×10^{-05}	8.54×10^{-05}
	Tertile2(16.80-20.80)	9	74	-0.81 (-1.15 ~ -0.46)	4.87×10^{-06}	3.90×10^{-05}
	Tertile1(<16.80)	57	75	ref		
LysoPC(18:1(9Z))	Continuous scale	68	223	-0.38 (-0.50 ~ -0.27)	1.38×10^{-10}	9.67×10^{-10}
	Tertile3(>7.90)	1	75	-1.66 (-2.53 ~ -0.78)	0.0002	0.0018
	Tertile2(6.37-7.90)	17	74	-0.44 (-0.72 ~ -0.15)	0.0026	0.0105
	Tertile1(<6.37)	50	74	ref		
Decanoylcarnitine	Continuous scale	68	223	-5.89 (-11.72 ~ -0.06)	0.0476	0.3332
	Tertile3(>0.37)	1	58	-0.91 (-1.84 ~ 0.02)	0.0554	0.2214
	Tertile2(0.28-0.37)	1	59	-0.98 (-1.91 ~ -0.04)	0.0413	0.3303
	Tertile1(<0.28)	8	59	ref		

Adjusted for age, sex, BMI, smoking, drinking.

Table S7. Results of pathway analysis between HC and RCC

Pathway Name	Match Status	*p*	-log(*p*)	Holm *p*	FDR	Impact	Matched metabolites
Alanine, aspartate and glutamate metabolism	5/28	1.08×10^{-42}	41.97	3.80×10^{-41}	3.8×10^{-41}	0.5345	L-Aspartic acid; L-Asparagine; L-Alanine; L-Glutamic acid; L-Glutamine
Arginine and proline metabolism	3/38	7.88×10^{-39}	38.10	$2.68 \times 10\text{-}^{37}$	1.4×10^{-37}	0.2576	4-Hydroxyproline; L-Glutamic acid; L-Ornithine
Arginine biosynthesis	4/14	3.96×10^{-38}	37.40	1.31×10^{-36}	4.6×10^{-37}	0.1777	L-Glutamic acid; L-Aspartic acid; L-Ornithine; L-Glutamine
Histidine metabolism	3/16	4.18×10^{-36}	35.38	1.34×10^{-34}	3.7×10^{-35}	0.2213	L-Glutamic acid; L-Histidine; L-Aspartic acid
Glycerophospholipid metabolism	2/36	4.16×10^{-30}	29.38	1.29×10^{-28}	2.9×10^{-29}	0.1118	Phosphatidylcholine(PC(14:0/16:0), PC(16:0/16:0), PC(14:0/18:2), PC(16:0/18:1), PC(16:0/18:2), PC(16:0/18:3), PC(14:0/20:4), PC(15:0/20:3), PC(15:0/20:4), PC(16:0/20:2), PC(16:0/20:3), PC(16:0/20:4), PC(16:0/20:5), PC(14:0/22:6), PC(15:0/22:4), PC(15:0/22:5), PC(15:0/22:6), PC(16:0/22:4), PC(16:0/22:5), PC(16:0/22:6), PC(18:0/22:6), PC(18:4/22:5), PC(20:1/22:6), PC(20:4/22:6)); 1-Acyl-sn-glycero-3-phosphocholine(LysoPC(16:0), LysoPC(16:1), LysoPC(17:0), LysoPC(18:0), LysoPC(18:1), LysoPC(18:2), LysoPC(20:3), LysoPC(20:4), LysoPC(22:6))
Glutathione metabolism	2/28	2.50×10^{-29}	28.60	7.49×10^{-28}	1.5×10^{-28}	0.0197	L-Glutamic acid; L-Ornithine
Aminoacyl-tRNA biosynthesis	12/48	3.67×10^{-26}	25.44	1.06×10^{-24}	1.8×10^{-25}	0.0000	L-Asparagine; L-Histidine; L-Phenylalanine; L-Glutamine; L-Aspartic acid; L-Methionine; L-Alanine; L-Lysine; L-Threonine; L-Tryptophan; L-Tyrosine; L-Glutamic acid
D-Glutamine and D-glutamate metabolism	2/6	3.93×10^{-25}	24.41	1.10×10^{-23}	1.4×10^{-24}	0.5000	L-Glutamic acid; L-Glutamine
Glyoxylate and dicarboxylate metabolism	2/32	3.93×10^{-25}	24.41	1.10×10^{-23}	1.4×10^{-24}	0.0000	L-Glutamic acid; L-Glutamine
Nitrogen metabolism	2/6	3.93×10^{-25}	24.41	1.10×10^{-23}	1.4×10^{-24}	0.0000	L-Glutamic acid; L-Glutamine
Tryptophan metabolism	1/41	6.32×10^{-25}	24.20	1.58×10^{-23}	2.0×10^{-24}	0.1431	L-Tryptophan
Butanoate metabolism	1/15	5.94×10^{-22}	21.23	1.43×10^{-20}	1.6×10^{-21}	0.0000	L-Glutamic acid
Porphyrin and chlorophyll metabolism	1/30	5.94×10^{-22}	21.23	1.43×10^{-20}	1.6×10^{-21}	0.0000	L-Glutamic acid
beta-Alanine metabolism	2/21	3.76×10^{-16}	15.43	8.27×10^{-15}	9.4×10^{-16}	0.0000	L-Aspartic acid; L-Histidine
Selenocompound metabolism	1/20	8.55×10^{-16}	15.07	1.79×10^{-14}	2.0×10^{-15}	0.0000	L-Alanine
Cysteine and methionine metabolism	1/33	7.39×10^{-15}	14.13	1.48×10^{-13}	1.6×10^{-14}	0.1045	L-Methionine
Glycine, serine and threonine metabolism	2/33	1.77×10^{-14}	13.75	3.35×10^{-13}	3.6×10^{-14}	0.0929	Sarcosine; L-Threonine
Lysine degradation	1/25	1.67×10^{-13}	12.78	3.01×10^{-12}	3.1×10^{-13}	0.0000	L-Lysine
Biotin metabolism	1/10	1.67×10^{-13}	12.78	3.01×10^{-12}	3.1×10^{-13}	0.0000	L-Lysine
Phenylalanine, tyrosine and tryptophan biosynthesis	2/4	8.44×10^{-12}	11.07	1.35×10^{-10}	1.4×10^{-11}	1.0000	L-Phenylalanine; L-Tyrosine
Phenylalanine metabolism	2/10	8.44×10^{-12}	11.07	1.35×10^{-10}	1.4×10^{-11}	0.3571	L-Phenylalanine; L-Tyrosine
Tyrosine metabolism	1/42	1.31×10^{-11}	10.88	1.83×10^{-10}	2.0×10^{-11}	0.1397	L-Tyrosine
Ubiquinone and other terpenoid-quinone biosynthesis	1/9	1.31×10^{-11}	10.88	1.83×10^{-10}	2.0×10^{-11}	0.0000	L-Tyrosine
Arachidonic acid metabolism	1/36	2.16×10^{-09}	8.666	2.59×10^{-08}	2.9×10^{-09}	0.0000	Phosphatidylcholine

Linoleic acid metabolism	1/5	2.16×10^{-09}	8.666	2.59×10^{-08}	2.9×10^{-09}	0.0000	Phosphatidylcholine
alpha-Linolenic acid metabolism	1/13	2.16×10^{-09}	8.666	2.59×10^{-08}	2.9×10^{-09}	0.0000	Phosphatidylcholine
Valine, leucine and isoleucine biosynthesis	1/8	7.59×10^{-08}	7.120	6.84×10^{-07}	9.8×10^{-08}	0.0000	L-Threonine
Sphingolipid metabolism	1/21	8.98×10^{-08}	7.047	7.18×10^{-07}	1.1×10^{-07}	0.0000	Sphingomyelin(SM(d18:1/16:0), SM(d18:1/18:0), SM(d18:1/20:0), SM(d18:0/20:2), SM(d18:1/22:0), SM(d18:1/24:1))
Purine metabolism	1/65	9.73×10^{-07}	6.012	6.81×10^{-06}	1.1×10^{-06}	0.0000	L-Glutamine
Pyrimidine metabolism	1/39	9.73×10^{-07}	6.012	6.81×10^{-06}	1.1×10^{-06}	0.0000	L-Glutamine
Nicotinate and nicotinamide metabolism	1/15	7.90×10^{-05}	4.102	0.0004	8.6×10^{-05}	0.0000	L-Aspartic acid
Pantothenate and CoA biosynthesis	1/19	7.90×10^{-05}	4.102	0.0004	8.6×10^{-05}	0.0000	L-Aspartic acid
Steroid biosynthesis	1/42	0.0016	2.790	0.0049	0.0017	0.0000	Cholesterol ester(CE(16:1)
Glycolysis / Gluconeogenesis	1/26	0.0088	2.054	0.0177	0.0091	0.0002	Triacylglycerol(TG(16:0/16:1/18:1), TG(16:0/16:1/18:2))
Glycerolipid metabolism	1/16	0.0216	1.665	0.0216	0.0216	0.0140	Triacylglycerol(TG(16:0/16:1/18:1), TG(16:0/16:1/18:2))

Metabolites missing from pathway analysis (m = 40) : L-Carnitine, Butyrylcarnitine, L-Octanoylcarnitine, 2-Octenoylcarnitine, Decanoylcarnitine, 9-Decenoylcarnitine, Asymmetric dimethylarginine, Creatinine, CE(18:2), D-Glucose, CE(18:3), DG(14:0/18:1/0:0), DG(18:0/24:1/0:0), DG(18:1/24:1/0:0), DG(20:2/24:1/0:0), TG(14:0/14:0/16:1), TG(16:1/14:0/16:1), PC(15:1/22:2), PC(21:0/22:2), PC(21:0/22:6), PC(o-16:1/16:1), PC(o-18:1/16:0), PC(o-16:0/18:2), PC(o-16:1/18:2), PC(o-18:1/18:2), PC(o-16:0/20:4), PC(P-16:0/20:4), PC(o-37:6), PC(P-18:0/20:4), PC(o-16:0/22:6), PC(o-18:0/22:6), PC(P-18:0/22:6), PC(o-40:8), SM(d18:1/14:0), SM(34:2), SM(d18:1/17:0), SM(39:1), SM(d17:1/24:0), SM(d17:1/24:1), SM(d18:1/24:0)

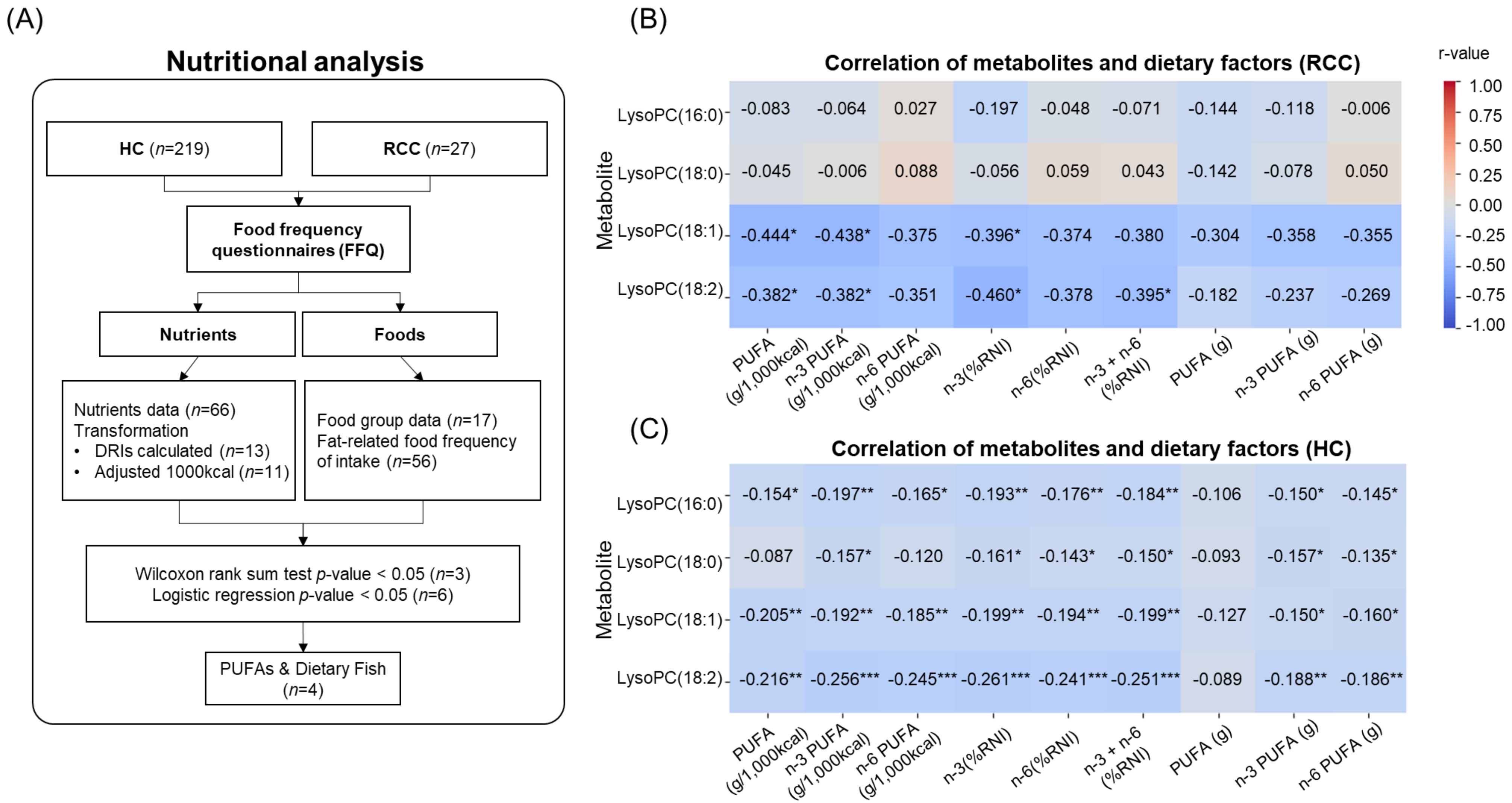

Figure S3. Correlation between LysoPC and PUFAs in each group and nutritional analysis flow chart.
LysoPC, Lysophosphatidylcholine, PUFA, polyunsaturated fatty acid. *$p<0.05$, **$p<0.01$, ***$p<0.001$
